# Moxibustion Reduces Ovarian Granulosa Cell Apoptosis Associated with Perimenopause in a Natural Aging Rat Model

**DOI:** 10.1155/2015/742914

**Published:** 2015-10-13

**Authors:** Xiao-Lan Shi, Chen Zhao, Shuai Yang, Xiao-Ying Hu, Shi-Min Liu

**Affiliations:** ^1^Department of Traditional Chinese Medicine, Shanghai Jiangwan Hospital, No. 22 Changzhong Road, Hongkou District, Shanghai 200434, China; ^2^School of Acupuncture and Tuina, Shanghai University of Traditional Chinese Medicine, Shanghai 201203, China

## Abstract

In recent years, concerns about the adverse effects of hormone replacement therapy have increased interest in alternative therapies for the management of the symptoms of perimenopause. Here, we investigated the effects of moxibustion, a traditional Chinese practice that is involved in heated *Artemisia vulgaris* (mugwort) stimulation, on hormonal imbalance and ovarian granulosa cell (GC) apoptosis in a rat model of perimenopause. Our results showed that mild warm moxibustion (MWM) modulated the circulating levels of estradiol and follicle-stimulating hormone and their receptors and inhibited apoptosis in the ovaries of perimenopausal rats, similar to the effect of estrogen. Further investigation revealed that the effects of MWM on ovary tissues and cultured GCs were mediated by the modulation of the activity of Forkhead box protein O1 and involved the JAK2/STAT3 pathway. Our results provide information on the factors and pathways modulated by MWM and shed light on the mechanism underlying the beneficial effect of moxibustion on the symptoms of perimenopause.

## 1. Introduction

Perimenopause refers to the period surrounding the transition into menopause, which is defined as the end of menstruation and fertility [1]. Menopause is characterized by the loss of estrogen production by the ovaries, which can have a wide range of effects on women's health and quality of life. The menopausal decrease in estrogen levels contributes to decreased bone mass and density, the accumulation and redistribution of adipose tissue, and an increased risk of metabolic disorders and cardiovascular disease [2–4]. Hormone replacement therapy, which was used widely in the 1960s and early 1970s, was found to be associated with an increased risk of endometrial and breast cancer, triggering an increased interest in alternative therapies for the treatment of perimenopause-related symptoms [5, 6].

Apoptosis is a critical mechanism for cellular homeostasis by which cells that are damaged, senescent, or no longer useful are eliminated via programmed cell death. During follicular growth and development, more than 99% of follicles undergo a degenerative process known as atresia that has been associated with apoptosis of granulosa cells (GCs) [7–9]. Although atretic follicles show the biochemical and morphological features of apoptosis, the exact molecular mechanisms and signal transduction pathways involved in the apoptosis of GCs remain unclear. The Forkhead box O (FOXO) family of transcription factors regulates the expression of genes associated with apoptosis, cell cycle progression, cellular homeostasis, and mitochondrial metabolism among others [10]. FOXO1 has been suggested to be involved in follicular development, and its levels and localization change during follicular atresia [11]. In GCs, follicle-stimulating hormone (FSH) functions as a survival factor by preventing apoptosis, whereas FOXO1 induces apoptosis and follicular atresia, and several signaling cascades are involved in the regulation of FOXO1 activity in response to FSH stimulation [12, 13]. In addition, FOXO1 has been suggested to be involved in the regulation of FSH and luteinizing hormone (LH) levels, whose interactions with ovarian regulatory factors are essential for GC proliferation, differentiation, and apoptosis [13, 14].

Moxibustion is a traditional Chinese therapy that consists of direct or indirect acupuncture-point stimulation using burned dried* Artemisia vulgaris *(mugwort). Moxibustion has been used for a wide range of conditions including arthritis, gastrointestinal disorders, gynecological complaints, and pain. However, clinical trial-based evidence supporting the effectiveness of moxibustion is limited [15].

In the present study, we examined the effects of moxibustion on ovarian GC apoptosis and explored the underlying mechanisms. Our results indicate that moxibustion inhibits the apoptosis of GCs via a mechanism involving FOXO1 and the Janus Kinase (JAK)/Signal Transducer and Activator of Transcription (STAT) pathway and reveal a potential novel mechanism modulating the effects of hormonal imbalances associated with perimenopause.

## 2. Materials and Methods

### 2.1. Animals

Immature female (3-4 weeks), young female (3-4 months), and perimenopausal female (11 months, weighting 180 ± 20 g) Sprague-Dawley rats were obtained from the Experimental Animal Center at the Shanghai Jiangwan Hospital, China. All rats were kept under controlled temperature (30 ± 2°C) and light (14 h light, 10 h dark) conditions with rat chow and water ad libitum. All experiments were approved by the Institutional Animal Research Ethics Committee of the Shanghai Jiangwan Hospital.

### 2.2. Groups and Treatment

Perimenopausal female (11 months) Sprague-Dawley rats were randomly divided into the following three groups (*n* = 10 per group): control, mild warm moxibustion (MWM), and conjugated equine estrogen (CEE) groups. In addition, 10 adult female rats (3-4 months) comprised the young group. Prior to the initiation of experiments, vaginal smears of the female rats were obtained at 8 : 30 am every morning to confirm the perimenopausal status of the older rats according to the estrous cycle. In the MWM group, moxa sticks were ignited 1-2 cm above Guanyuan (CV4) and Shenshu (BL23) for 20 min once daily for 6 days with 1 rest day, and the temperature of the local area was kept at 43 ± 2°C. The BL23 acupoints on both sides were used interchangeably. The CEE group received 0.1 mg/kg/d CEE (Wyeth Pharmaceuticals Inc., Lotio 071207) by gavage administration once a day. The rats in the control and young groups did not receive any treatment. Five rats from each group were randomly blooded from the aorta abdominalis and then sacrificed after 4 and 8 weeks, and their ovaries and uterus were removed.

### 2.3. Radioimmunoassay (RIA) for Estradiol, FSH, and LH

The collected blood samples were coagulated for 2 h and centrifuged at 4°C, 4000 rpm, for 10 min, to separate the serum. One part of the separated serum was used for the measurement of estradiol (*E*_2_), FSH, and LH and stored at −80°C until radioimmunoassay according to the kit manuals. *E*_2_, LH, and FSH kits were purchased from Beijing Kemei biotechnology Co., Ltd. (Lotio 081025). Other samples were stored at −20°C for culturing GCs* in vitro*.

### 2.4. TUNEL Assay

TUNEL staining was performed using a fluorescence detection kit (Roche, Indianapolis, IN, USA). Paraffin embedded sections (5 *μ*m) were stained with terminal deoxynucleotidyl transferase and fluorescein-dUTP after proteinase K treatment and incubated for 60 min at 37°C in a humidified chamber in the dark. Sections were rinsed three times with PBS and visualized with a fluorescence microscope (Olympus, Tokyo, Japan) equipped with a digital camera. The percentage of apoptotic cells was determined in micrographs of TUNEL positive and DAPI-stained nuclei using Image J software in 10 random fields at 400x magnification. At least 100 cells were counted in each field.

### 2.5. Immunohistochemistry

For immunohistochemistry, paraffin embedded sections (5 *μ*m) of the ovaries of rats in the different groups were deparaffinized, treated with 1% H_2_O_2_ in methanol for 30 min to block endogenous peroxidase activity, washed in Tris-buffered saline, blocked with 10% normal rabbit serum, and incubated overnight at 4°C with rabbit-anti-human polyclonal antibodies against Bcl-2, Bax (Santa Cruz Biotechnology, Santa, CA, USA), and active caspase-3 (Abcam, Cambridge, MA, USA). After washing in TBS, slides were incubated with biotinylated goat anti-rabbit IgG in TBS with 0.05% BSA. Sections were then washed in TBS and incubated with avidin and biotin (Vectastain ABC-Elite kit, Vector Laboratories, Burlingame, CA, USA), and bound antibody was visualized after the addition of a solution of 3,3′-diaminobenzidine tetrachloride (DAB, Sigma, St. Louis, MO, USA) in Tris-HCl with 0.03% H_2_O_2_. DAPI staining for the cell nucleus was used to calculate the total number of cells. The stage of each section was scored based on THE staining density and the percentage of positively stained cells. The mean staining intensity for each slice was calculated. The intensity of immunostaining was statistically analyzed using semiquantitative scores of “1” for weak or no staining; “2” for mild staining; “3” for moderate staining; and “4” for strong staining when, respectively, <1, 1–10, 10–50, and >50 percent of the cells in the follicular or corpus luteum cross section stained positively.

### 2.6. Granulosa Cell Isolation and Culture

Rats (female, 3-4 weeks) were injected intraperitoneally with 60 units of pregnant mare serum gonadotropin (PMSG, Animal Drugs Factory, Huangzhou, Zhejiang, China). After 48 h, the rats were sacrificed, the ovaries were removed, and GCs were isolated by needle puncture under an inverted microscope. After obtaining single cell suspensions, they were washed twice with PBS and cultured with complete medium, including DMEM/F12 (1 : 1) medium supplemented with 10% FBS, 100 U/mL penicillin, and 100 U/mL streptomycin at 37°C and 5% CO_2_.

GCs were plated in 24-well cultures dishes with complete medium overnight. The medium was removed and replaced with the culture media supplemented with 10% of the separated sera from the different natural aging model groups (control, CEE, and MWM) and the young group at 8 weeks, separately. Validating the effects of MWM on cultured GCs was mediated by the modulation of the activity of FOXO1, before treatment with the MWM serum, and GCs were transfected with constructs containing the full length open reading frame of mouse FOXO1 (OriGene) to overexpress FOXO1. The cells were cultured at 37°C and 5% CO_2_ for 48 h, and cells were collected for apoptosis rate analysis and western blot analysis.

### 2.7. Apoptosis Analysis by Flow Cytometry

Cells in suspension were washed in ice-cold PBS and incubated at 4°C for 5 min. After removal of the supernatant, cells were filtered with ice-cold binding buffer to a concentration of 5 × 10^5^–5 × 10^6^/mL. A 100 *μ*L cell suspension was treated with 5 *μ*L of Annexin V-FITC (100 U/mL, Sigma) and 2.5 *μ*L of propidium iodide (50 *μ*g/mL, Sigma), mixed, and incubated on ice in the dark for 10 min. Then, 400 *μ*L of ice-cold 1x binding buffer was added to the suspension, mixed for 30 min, and analyzed by flow cytometry using a FACSCalibur flow cytometer (Becton-Dickinson, San Jose, CA, USA).

### 2.8. Western Blot Analysis

Total lysates were prepared in radioimmune precipitation assay buffer and protein content was determined using the BCA method (Pierce, Rockford, IL, USA). Protein samples containing 15 *μ*g of protein were separated in 7.5% SDS-polyacrylamide gels and transferred to PVDF membranes (Millipore, Billerica, MA). Membranes were blocked for 2 h in 5% milk powder in PBS and then incubated in primary antibodies against ER, FSHR, LHR, Bcl-2, Bax, p-JAK2, JAK2, p-STAT3, STAT3, *β*-actin (Santa Cruz Biotechnology), active caspase-3, FOXO1, and p-FOXO1 (Cell Signaling Technology, Beverly, MA) overnight at 4°C followed by HRP-conjugated secondary antibodies for 2 h at room temperature. After washing, proteins were detected using SuperSignal West Pico Chemiluminescent Substrate (Pierce). *β*-actin was used as the loading control.

### 2.9. Statistical Analysis

Results are expressed as the mean ± SD. Differences were analyzed using one-way analysis of variance (ANOVA) or *t*-test. The level of significance was set at *p* < 0.05. All experiments were repeated at least three times.

## 3. Results

### 3.1. Moxibustion Regulates the Levels of Circulating *E*_2_, FSH, and LH in Aged Perimenopausal Rats

**Figure 1:**
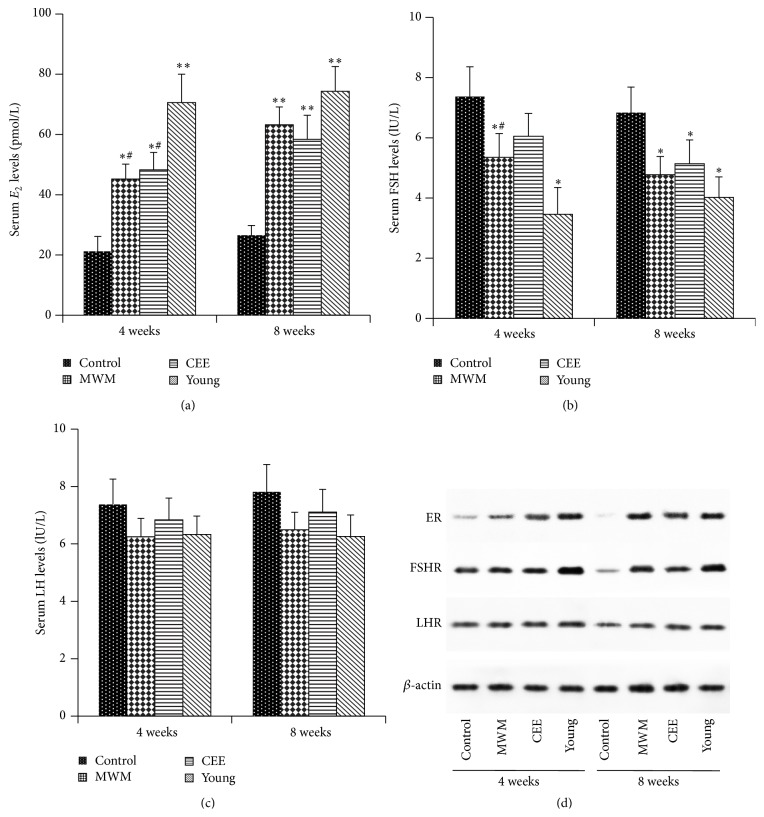
Serum levels of *E*_2_, FSH, and LH and expression of the corresponding receptors. Serum levels of *E*_2_ (a), FSH (b), and LH (c) were measured in control, MWM, CEE, and young groups at 4 and 8 weeks by radioimmunoassay. Data are presented as the mean ± standard error of the mean (*n* = 5 rats/group, 3 repetitions). *∗*: versus control group, #: versus young group; ^*∗*, #^ *p* < 0.05, ^*∗∗*^ *p* < 0.01.

Serum levels of *E*_2_, FSH, and LH were measured in control perimenopausal rats treated with or without MWM and CEE and compared with those in the young (3-4 months old) group. Serum *E*_2_ levels were significantly lower in control than in young rats, and treatment with MWM significantly increased *E*_2_ levels at 4 and 8 weeks in perimenopausal rats similar to the effect of CEE (Figure 1(a)). FSH levels were significantly decreased by MWM compared to those in the control group at 4 and 8 weeks, and CEE had a similar effect, although the decrease was only significant at 8 weeks (Figure 1(b)). MWM and CEE did not significantly affect the serum levels of LH in the aged group at 4 and 8 weeks (Figure 1(c)). Western blot analysis of the expression of the receptors for estrogen, FSH, and LH (ER, FSHR, and LHR) in the uterus of rats in the different groups showed that MWM and CEE upregulated the expression of ER and FSHR compared to the control (Figure 1(d)). At 4 weeks, FSHR expression was slightly increased in the MWM and CEE groups, whereas, at 8 weeks, MWM markedly increased ER expression compared to the control and CEE groups. LHR expression levels did not differ among the four groups of rats (Figure 1(d)). The changes in the expression levels of ER, FSH, and LHR were consistent with the observed hormonal changes and indicate that moxibustion modulates female hormones and their receptors in aged perimenopausal rats.

### 3.2. Moxibustion Reduces Apoptosis in the Ovaries of Aged Perimenopausal Rats

**Figure 2:**
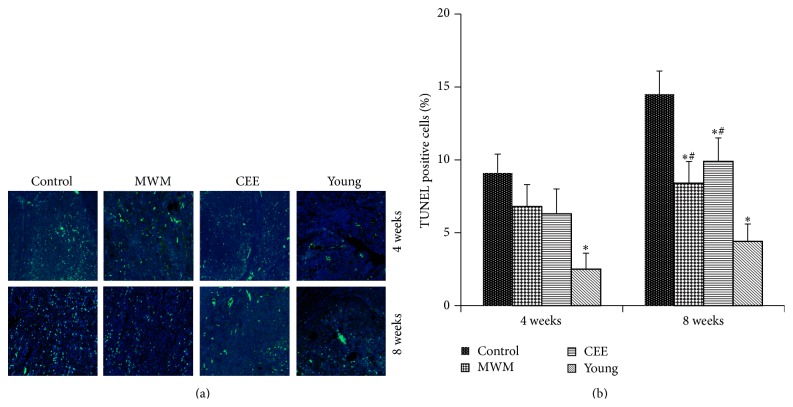
Effects of moxibustion on apoptosis in an* in vivo* model of perimenopause. (a) Representative images of TUNEL staining in rat ovarian tissues obtained from control, MWM, CEE, and young groups 4 and 8 weeks after the intervention (200x). TUNEL positive nuclei were visualized with fluorescein (green). Nuclei were stained with DAPI and are shown in blue. (b) Quantification of TUNEL positive cells. Data are expressed as the mean ± SD; ^*∗*^ *p* < 0.05 versus control group; ^#^ *p* < 0.05 versus young group.

To examine the effect of moxibustion on cell apoptosis associated with perimenopause, ovarian tissues were obtained from middle-aged rats treated as indicated and cell apoptosis was evaluated using TUNEL staining.  Figure 2(a) shows representative images of TUNEL staining in ovarian tissues of control, MWM-treated, CEE-treated, and young rats at 4 and 8 weeks after treatment, with green fluorescence indicating TUNEL positive nuclei. Analysis of staining intensity showed that apoptosis rates were higher in the natural aged perimenopausal rats than the young rats at both time points. MWM and CEE treatment slightly decreased the rate of apoptosis at 4 weeks and significantly decreased the number of TUNEL positive nuclei at 8 weeks in the ovaries of perimenopausal rats (*p* < 0.05) (Figure 2(b)).

**Figure 3:**
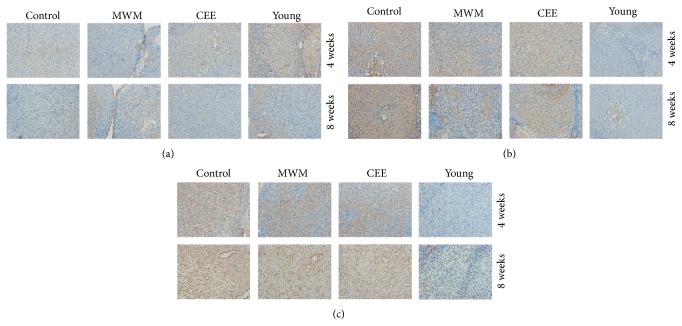
Immunohistochemical staining of apoptosis-related proteins in ovarian tissues. (a)–(c) Representative images of immunostaining of ovary tissues of rats in the control, MWM, CEE, and young groups against Bcl-2 (a), Bax (b), and caspase-3 (c). The antiapoptotic protein Bcl-2 shows higher staining intensity, whereas Bax and caspase-3 show lower staining intensity in MWM and CEE than in the controls at 8 weeks.

Immunohistochemical analysis of the expression of the antiapoptotic protein Bcl-2 and the proapoptotic proteins Bax and caspase-3 showed that MWM and CEE upregulated the expression of Bcl-2 and downregulated that of Bax and caspase-3 in ovarian tissues of perimenopausal rats compared to the levels in untreated controls, with obvious differences observed at 8 weeks (Figure 3).  Table 1 summarizes the relative immunostaining intensity of each gene.

### 3.3. Involvement of FOXO1 and the JAK2/STAT3 Pathway in GC Apoptosis Associated with Perimenopause

**Figure 4:**
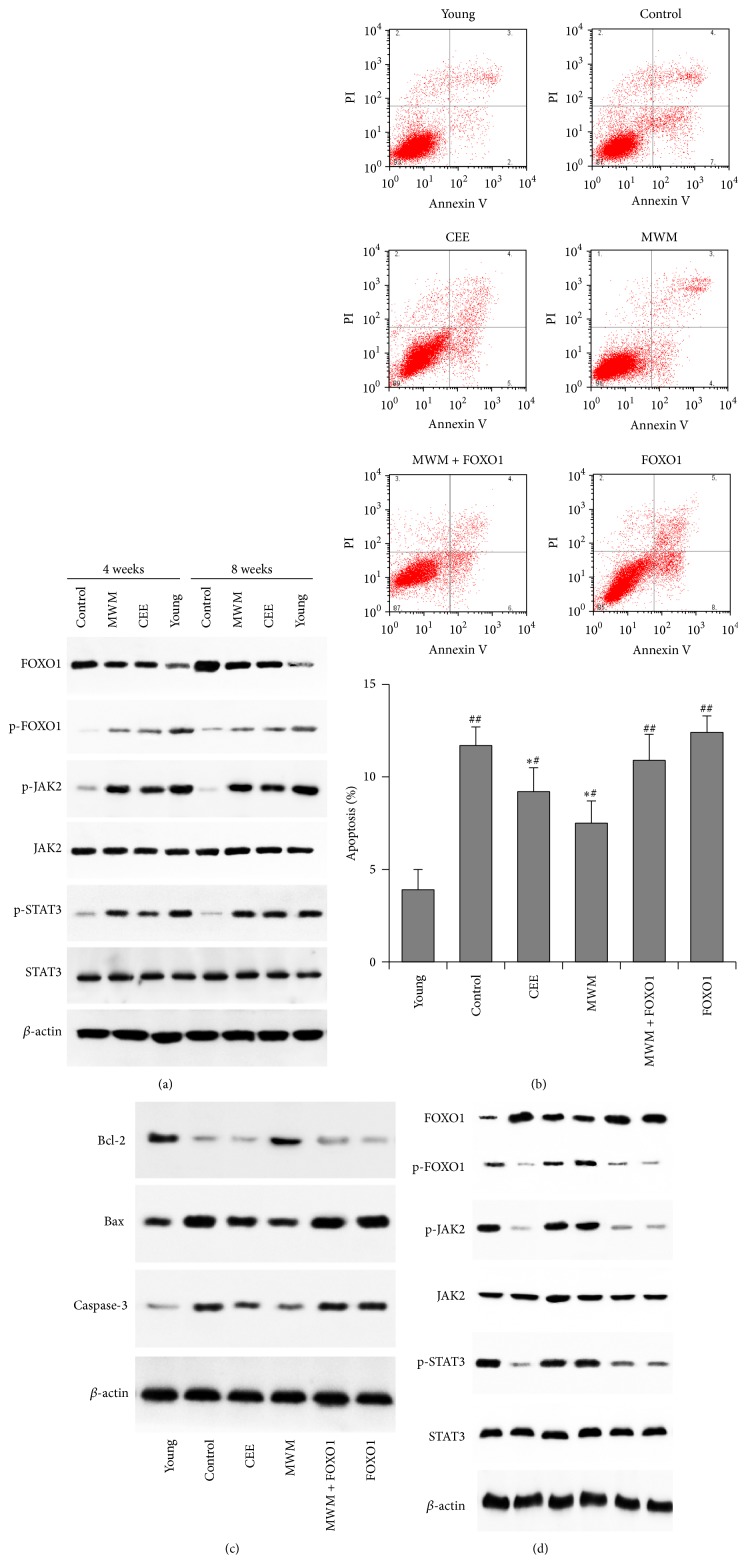
Involvement of FOXO1 in GC apoptosis associated with perimenopause. (a) Western blot detection of FOXO1, p-FOXO1, p-JAK2, JAK2, p-STAT3, and STAT3 protein levels in ovary tissues of natural aged perimenopausal rats. MWM downregulated FOXO1 and upregulated p-FOXO and activated the JAK2/STAT3 pathway. (b)–(d) GCs were cultured in DMEM/F12 medium containing 10% serum from the different perimenopausal model groups and young rats. (b) Effect of MWM and FOXO1 overexpression on GC apoptosis induced by natural aging rat serum assessed by flow cytometry. The rate of GC apoptosis was expressed as the mean ± SD. *∗*: versus control group, #: versus young group; ^*∗*, #^ *p* < 0.05; ^##^ *p* < 0.01. (c) Western blots analysis of the antiapoptotic protein Bcl-2 and the proapoptotic proteins Bax and caspase-3. (d) Western blot analysis of FOXO1, p-FOXO1, and JAK/STAT pathway protein levels. *β*-actin was used as the positive (loading) control. Representative images from three independent experiments are shown.

To explore the mechanisms underlying the MWM-induced inhibition of apoptosis in the ovary, the expression and activation of FOXO1, JAK2, and STAT3 were examined in the ovaries of rats in the different treatment groups. STATs are transcription factors that are activated in response to cytokines and growth factors, and activation of STAT3 is associated with cell proliferation and the inhibition of apoptosis [16]. Phospho-JAK2 phosphorylates STAT3, which translocates to the nucleus to regulate the expression of several genes. The JAK2/STAT3 pathway interacts with FOXO1 via the phosphoinositol 3 kinase (PI3K)/Akt pathway, and dephosphorylation and nuclear translocation of FOXO1 induce apoptosis. Western blot analysis showed that FOXO1 levels were higher in the ovary tissues of natural aged perimenopausal rats than in those of young rats (Figure 4(a)). MWM and CEE markedly increased the levels of p-FOXO1 and downregulated FOXO1 expression at 4 and 8 weeks and increased the levels of phospho-JAK2 and phospho-STAT3 at 4 and 8 weeks, confirming the antiapoptotic effect of MWM in the ovaries of perimenopausal rats. In GCs cultured in medium supplemented with serum from rats in the different groups, FOXO1 overexpression reversed the effect of MWM on the inhibition of apoptosis (Figure 4(b)). Western blot analysis showed that the upregulation of Bcl-2 and the downregulation of Bax and caspase-3 induced by MWM were reversed by FOXO1 overexpression (Figure 4(c)). Similarly, FOXO1 overexpression reversed the effect of MWM on the activation of JAK2 and STAT3 phosphorylation in GCs from perimenopausal rats (Figure 4(d)). Taken together, these results indicate that the effect of MWM on the inhibition of apoptosis in GCs is mediated by the modulation of pro- and antiapoptotic proteins by the FOXO1/JAK2/STAT3 axis.

## 4. Discussion

Follicular growth and atresia in the ovary are regulated by complex interactions between hormones, steroids, growth factors, cytokines, and proteins [17]. GCs play an essential role in follicular maintenance and atresia, as they provide molecules essential for follicular growth, and their self-killing by apoptosis is a central process leading to follicular atresia. The fate of GCs is regulated by interactions between gonadotropins (FSH and LH), and the activity of FSH in the ovaries has been shown to be partially mediated by FOXO1, suggesting that the role of GCs in the ovary involves a network of interacting molecules that includes FSH and FOXO1 [13]. In the present study, we examined the mechanism underlying the effect of MWM on the hormonal imbalance of perimenopause and elucidated a potential underlying mechanism involving FOXO1 and the JAK2/STAT3 pathway.

Many of the physical and psychological symptoms of perimenopause are attributed to the depletion of estrogen and the associated hormonal imbalance. Traditional Chinese medicine has been used for a long time for the treatment of gynecological disorders, and an increasing number of studies have focused on elucidating the pharmacological effects of herbal medicines. Here, we determined the effect of MWM on the serum levels of *E*_2_, FSH, and LH in a natural aging rat model and showed that MWM increased the circulating levels of *E*_2_ and decreased those of FSH without affecting the levels of LH, similar to the effects of CEE. Similar effects on *E*_2_/FSH/LH levels were obtained in previous studies using a preparation of herbal medicines designated as RRF, which restored hormonal balance, alleviated reproductive organ and skeleton degeneration, and restored bone mineral density in a rat model [18, 19]. On the other hand, Danggui Buxue Tang (DBT), a traditional Chinese preparation used widely to alleviate perimenopausal symptoms, exhibits weak estrogenic properties and its activity was shown to be largely independent of the estrogen receptor in* in vivo* experiments [20]. Our present results showed that MWM upregulated the expression of the receptors for estrogen and FSH similar to the effect of CEE, suggesting that its function in the uterus is ER dependent. However, research on the mechanisms underlying the various effects of MWM is limited; therefore, further investigation of the effect of MWM on the different factors regulating follicular growth and atresia is warranted.

MWM inhibited apoptosis concomitant with the upregulation of antiapoptotic proteins and the downregulation of proapoptotic proteins in our natural aged rat model, similar to the effect of CEE. Moxibustion was previously shown to inhibit epithelial cell apoptosis by modulating Bcl-2/Bax expression in a rat model of ulcerative colitis [21]. In a rat model of Crohn's disease, moxibustion inhibited colonic epithelial cell apoptosis by downregulating tumor necrosis factor- (TNF-) alpha and TNF receptor-1 [22]. In the present study, further investigation revealed the involvement of FOXO1 and the JAK2/STAT3 pathway in the antiapoptotic effect of MWM in the ovary and the direct inhibition of GC apoptosis. FOXO1 is highly expressed in GCs, where its activity is modulated by steroid hormones [23]. FOXO1 activity is partially regulated by its Akt-mediated phosphorylation, which protects cells from apoptosis by inhibiting its transactivation of proapoptotic genes [24]. In the present study, MWM increased the levels of phosphorylated FOXO1 in ovary tissues in our aged rat model, suggesting that the effect of MWM is mediated by the regulation of FOXO1 activity. This was supported by the reversal of MWM-induced inhibition of apoptosis by FOXO1 overexpression in cultured GCs. The changes in the activation status of FOXO1 induced by MWM were accompanied by the upregulation of phospho-JAK2 and phospho-STAT3, and this effect was also reversed by FOXO1 overexpression indicating the involvement of the JAK2/STAT3 pathway in the effect of MWM via FOXO1.

FSH, which is required for the production of estrogen and the development of antral follicles, was shown to inhibit apoptosis in GCs via a mechanism involving FOXO1 [13]. The responses to FSH are achieved through the activation of several signaling cascades in GCs, including protein kinase A (PKA), protein kinase B (PKB/AKT), p38 mitogen-activated protein kinase (p38-MAPK), and extracellular signal-regulated kinases 1 and 2 (ERK1/2), which are involved in the regulation of FOXO1 activity. Our results showed that MWM modulated the levels of FSH and inhibited apoptosis via a mechanism involving FOXO1. MWM decreased the levels of circulating FSH and upregulated the expression of FSHR in the uterus, while decreasing the activity of FOXO1. However, the involvement of the PI3K/Akt pathway in modulating the activity of FOXO1 was not assessed. Therefore, the effect of MWM on FOXO1-dependent apoptosis mediated by the effect of FSH on the PI3K/AKT kinase cascade should be investigated further.

**Figure 5:**
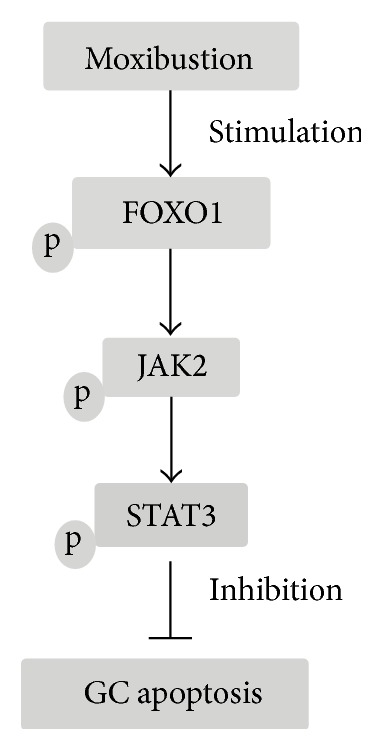
Role of FOXO1/JAK2/STAT3 pathway in the regulation of perimenopausal rats. MWM inhibition of apoptosis in GCs in a natural aging perimenopausal rat model, the JAK2/STAT3 pathway involved in the effect of MWM via FOXO1.

In conclusion, the present study examined the effect of moxibustion on GC apoptosis and its relation to hormonal imbalance in a natural aging perimenopausal rat model. Our results suggested that MWM inhibits GC apoptosis via a mechanism involving FOXO1 and the JAK2/STAT3 pathway. The schematic of this pathway was shown in Figure 5. However, further investigation is necessary to clarify the factors and pathways regulated by MWM, which would shed light on the mechanisms underlying the beneficial effects of moxibustion on the symptoms of perimenopause.

## Figures and Tables

**Table 1:** Relative intensity of positive immunostaining of each gene in the rat ovary.

	Bcl-2	Bax	Caspase-3
4 weeks			
Control	1~2	3~4	3~4
MWM	1~2	2~3	3
CEE	1~2	2~4	3
Young	2~3	1	1
8 weeks			
Control	1	4~3	4
MWM	2	3	3
CEE	1~2	3~4	3~4
Young	2	1~2	1~2

1: weak or no staining; 2: mild staining; 3: moderate staining; 4: strong staining.
